# Preparation and Comprehensive Evaluation of the Efficacy and Safety of Chlorantraniliprole Nanosuspension

**DOI:** 10.3390/toxics12010078

**Published:** 2024-01-16

**Authors:** Xiquan Ding, Liang Guo, Qian Du, Tingyu Wang, Zhanghua Zeng, Yan Wang, Haixin Cui, Fei Gao, Bo Cui

**Affiliations:** 1 Institute of Environment and Sustainable Development in Agriculture, Chinese Academy of Agricultural Sciences, Beijing 100081, China; 2 College of Pharmacy, Henan University of Chinese Medicine, Zhengzhou 450046, China

**Keywords:** chlorantraniliprole, nanosuspension, efficacy, safety

## Abstract

Chlorantraniliprole is a broad-spectrum insecticide that has been widely used to control pests in rice fields. Limited by its low solubility in both water and organic solvents, the development of highly efficient and environmentally friendly chlorantraniliprole formulations remains challenging. In this study, a low-cost and scalable wet media milling technique was successfully employed to prepare a chlorantraniliprole nanosuspension. The average particle size of the extremely stable nanosuspension was 56 nm. Compared to a commercial suspension concentrate (SC), the nanosuspension exhibited superior dispersibility, as well as superior foliar wetting and retention performances, which further enhanced its bioavailability against *Cnaphalocrocis medinalis*. The nanosuspension dosage could be reduced by about 40% while maintaining a comparable efficacy to that of the SC. In addition, the chlorantraniliprole nanosuspension showed lower residual properties, a lower toxicity to non-target zebrafish, and a smaller effect on rice quality, which is conducive to improving food safety and the ecological safety of pesticide formulations. In this work, a novel pesticide-reduction strategy is proposed, and theoretical and data-based support is provided for the efficient and safe application of nanopesticides.

## 1. Introduction

**Figure 1:**
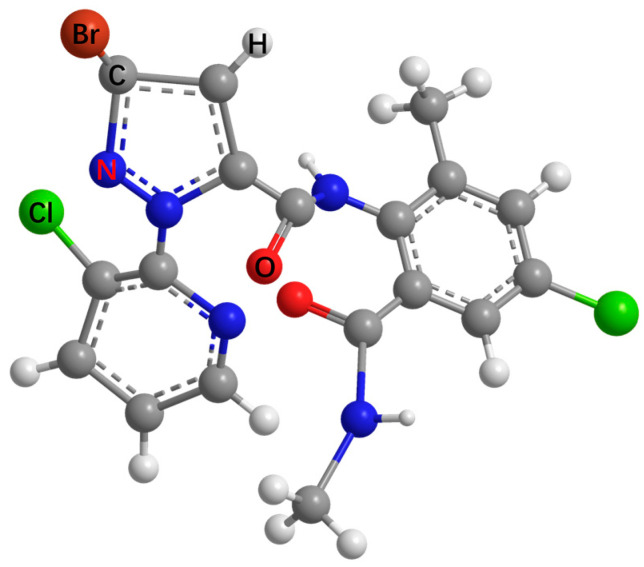
Stereochemical structure of chlorantraniliprole.

Chlorantraniliprole is an anthranilic diamide insecticide (Figure 1) that has been widely used in rice fields to control *Cnaphalocrocis medinalis, Nilaparvata lugens Stal, Chilo suppressalis*, and other pests. It has both contact and stomach poison activities [1,2]. The insecticidal mechanism of chlorantraniliprole involves the activation of the ryanodine receptor, leading to the discharge of intracellular calcium in insects’ smooth and striated muscle cells, which causes muscular regulation weakness and paralysis, culminating in pests’ deaths [3,4]. However, the low solubility of chlorantraniliprole in both water (1.023 mg/L) and organic solvents (3.446 mg/L in acetone, 1.714 mg/L in methanol, 0.711 mg/L in acetonitrile, 1.144 mg/L in ethyl acetate at 20–25 °C) has greatly hindered the development of highly efficient and environmentally friendly formulations. Nowadays, chlorantraniliprole is mainly formulated as a suspension concentrate (SC) and water-dispersible granule (WDG). For conventional formulations, dust drift and roll down result in the loss of >50% of the applied pesticides during field application [5,6]. In addition, organic solvents and excessive amounts of emulsifiers are used to maintain effective formulation performance. The low efficacy and the composition of conventional formulations lead to resource waste, pest resistance, and a series of food safety and environmental pollution issues [7,8,9]. Therefore, there is an urgent need to develop a green and scalable strategy to build an environmentally friendly chlorantraniliprole delivery system that is highly efficacious and capable of tackling the problems related to the use of sparingly soluble pesticides.

According to the Noyes–Whitney equation, a reduction in particle size can increase the saturation solubility and dissolution rate of materials [9], thereby improving the dispersibility of poorly soluble pesticide compounds. It has been widely demonstrated that nanotechnology can enhance the solubility, dispersibility, targeting, bioavailability, and safety of sparingly soluble pesticides, which has caused them to attract significant interest in the design and construction of nanopesticides [10,11,12,13]. Currently, there are two methods for preparing nanopesticides, namely, bottom-up and top-down approaches. Bottom-up approaches, such as micro-precipitation and solvent evaporation, involves the aggregation of molecules into nanoparticles [14,15]. Top-down approaches rely on mechanical forces to process larger particles into nanoscale particles. The typical top-down processes are media milling, high-pressure homogenization, dry co-grinding, and melt emulsification [16,17,18,19,20,21,22,23]. Patel et al. concluded that top-down techniques could significantly reduce drug particle sizes, increase their specific surface areas, and accelerate their dissolution rates, with their final formulations exhibiting good physical stability compared to conventional formulations [23]. Chin et al. produced a carbendazim nanosuspension via media milling which exhibited a superior stability and a 13.5% increase in insecticidal efficiency over a micron-sized suspension [24]. Francesco et al. prepared a zoxamid nanosuspension using the same method and increased the solubility 1.6-fold compared to the SC formulation while improving the deposition and retention abilities of the active ingredient on tomato plants [25]. In our previous work, we successfully constructed lambda–cyhalothrin and abamectin nanosuspensions using melt emulsification–high-pressure homogenization and wet milling methods, proving the effectiveness of nanotechnology for enhancing the dispersibility and biological activity of poorly soluble pesticides [26,27,28].

It is imperative to emphasize that recent studies on nanopesticides primarily concern formulation characterization and biological activity evaluations, and there is still a relative paucity of potential risk assessments on target and non-target organisms. In the mid-20th century, the widespread use of organochlorine pesticides such as DDT and lindane led to severe air, soil, and water pollution and caused the death of plants and animals through accumulation effects. Studies have linked the increasing incidence of nervous system damage like Alzheimer’s disease, cardiovascular diseases, and diabetes to the consumption of pesticide-contaminated food [29,30]. Some types of pesticides are carcinogenic and mutagenic, seriously affecting human genes and causing harm to the growth and development of future generations [31]. Nanopesticides are likely to settle in water and remain in sediments, causing toxicity to aquatic organisms, and to accumulate in fish through ingestion, thus affecting human health through the food chain [32,33]. In addition, pesticides may damage the nutritional value of crops, reducing the mineral content and inhibiting synthesis of proteins, sugars, and vitamins [34]. Rico et al. found that CeO_2_ nanoparticles can reduce the content of two essential elements (Fe and S), proteins (prolamin and glutelin), fatty acids (lauric and valeric acids), and starch in three rice varieties (high-, medium-, and low-amylose) [35]. Zhao et al. found that thifluzamide can change the chlorophyll, phenol, flavonoid, and protein contents in rice plants and that thifluzamide-loaded mesoporous silica nanoparticles could relieve the damage caused by thifluzamide to rice seedlings [36]. However, there are still relatively few reports focusing on both the nanopesticide residues and their impacts on food quality.

It is crucial to systematically evaluate the effect of nanopesticides on non-target organisms and foodstuffs while improving the effectiveness of pesticides. The potential environmental risk is a key issue, constraining the development and application of novel pesticide nanoformulations as well as the improvement of pesticide regulatory regimes. In this study, a facile and low-cost media milling technique was employed to prepare a chlorantraniliprole nanosuspension. The nanosuspensions’ physicochemical properties and field efficacy were systematically compared with a conventional SC to reveal the enhancing effect of the nanoformulation and its underlying mechanism. In particular, in this study, we also focused on nanopesticides’ safety issues from multiple perspectives related to pesticide residues in rice leaves and grains, effects on rice quality, and toxicity to non-target zebrafish. We propose a pesticide-reduction strategy and provide a theoretical basis and technical support for the efficient and safe application of nanopesticides.

## 2. Results and Discussion

### 2.1. Optimization of the Preparation Parameters of the Chlorantraniliprole Nanosuspension

According to our previous research, a composite surfactant composed of MRES and polycarboxylate (1:1, *w*/*w*) can provide effective electrostatic repulsion and a steric stabilization effect to stabilize the chlorantraniliprole nanoparticles produced by high-pressure homogenization combined with lyophilization. Therefore, the same surfactant combination was also applied for the preparation of the chlorantraniliprole nanosuspension by wet media milling. The surfactant content affects the amount of adsorption on the surface of the poor-water-soluble pesticide and further influences the formulation’s dispersibility and stability. As shown in Table 1, an increase in surfactant content can reduce the particle size to a certain extent, but this effect was no longer evident when the surfactant amount was 50%. Therefore, a 1:2 (*w*/*w*) surfactant/pesticide ratio was used to prepare the chlorantraniliprole nanosuspension. In addition, the milling parameters, especially the milling time, have a crucial impact on the particle size and distribution of the resultant suspensions. As shown in Table 1, the milling time significantly affected the particle sizes of the chlorantraniliprole nanosuspensions, although it had essentially no effect on the PDI value. As the milling time increased from 0.5 h to 1 h, the average particle size of the chlorantraniliprole nanosuspension decreased from 288.4 nm to 244.2 nm, and when the milling time increased to 2 h, the particle size continued to decrease to 161.9 nm but increased again to 181.0 nm as the time was extended to 3 h. The reason for this is that a short milling period does not provide sufficient energy to break up large particles, while excessive milling generates a large amount of heat and induces particle agglomeration. Hence, 2 h was chosen as the optimal milling time for synthesizing the chlorantraniliprole nanosuspension.

### 2.2. Particle Size and Zeta Potential

Particle size, distribution, and zeta potential are crucial indicators for evaluating the stability of water-based formulations. PDI values lower than 0.3 suggest a narrow size distribution, and a smaller PDI signifies a more uniform system. PDI values higher than 0.3 indicate that the system is susceptible to destabilization phenomena like Ostwald ripening and sedimentation [37,38]. As depicted in Table 2, the average particle size and PDI of the chlorantraniliprole nanosuspension were 161.9 ± 0.2 nm and 0.186 ± 0.010, respectively, measured using dynamic light scattering. In contrast, the particle size and PDI of the conventional SC formulation were 678.7 ± 27.3 nm and 0.446 ± 0.021, respectively, 4.2 and 2.4 times higher than that of the nanosuspension. Following the Ostwald Freundlich and Noyes Whitney equations, as the particle size decreases, the saturation solubility and dissolution rate of substances increase [39,40,41]. Consequently, the nanosuspension is more conducive to dissolving and dispersing poorly soluble pesticides in water, thereby mitigating the concentration discrepancies and reducing the incidences of chemical damage during spraying.

The potential polarity hinges upon the nature of the charge at the particle interface and is closely linked to the type and quantity of surfactants. The zeta potential of the chlorantraniliprole nanosuspension was measured to be −43.1 ± 0.7 mV, while the value of SC was −29.4 ± 0.3 mV (Table 2). Typically, an absolute zeta potential greater than 30 mV is indicative of system stability [42]. In this investigation, the highly negative zeta potential of the nanosuspension demonstrates that the anionic surfactants were indeed adsorbed on the pesticide surface and provided strong electrostatic repulsion against aggregation, thus enhancing the system stability [43].

### 2.3. Morphology

**Figure 2:**
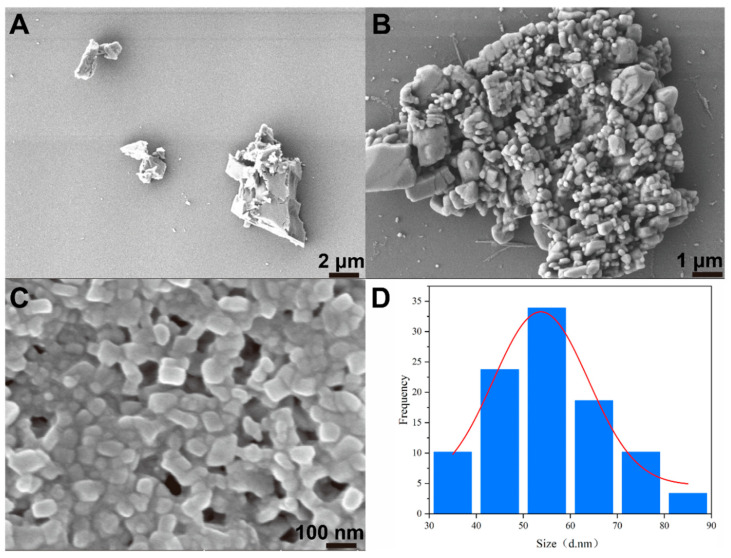
Morphologies of the chlorantraniliprole (**A**) TC, (**B**) SC, and (**C**) nanosuspension. (**D**) The statistical particle size of the nanosuspension (based on SEM images).

The morphologies of the chlorantraniliprole TC, SC, and nanosuspension are presented in Figure 2A–C. Due to the poor solubility and dispersibility of chlorantraniliprole in water, the TC exhibited an irregular blocky structure with micron-sized dimensions. Although the SC particle size was lower than that of the TC, it was still unevenly distributed, with micron and nanoparticles co-existing and noticeable aggregation. The suspension is a thermodynamically unstable system, and its long-term stabilization is an uphill battle against thermodynamics [41]. According to the Gibbs–Thomson equation [6,41] and the principle of thermodynamic energy reduction, the chlorantraniliprole SC with a broad size distribution is prone to undergo Ostwald ripening, leading to clarification, sedimentation, and agglomeration [44]. In contrast, the chlorantraniliprole nanosuspension produced through wet media milling exhibited a uniform and regular block-like structure. Based on SEM images, we found that the statistical particle size ranged from 34 nm to 86 nm, with an average diameter of 56 nm (Figure 2D). This value was smaller than that measured by DLS. This difference is due to the fact that SEM reflects the monodispersed particle size in a dried state, whereas DLS reflects the hydrodynamic diameter with hydrated or diffuse layers on the particle periphery [45]. Additionally, larger particles or aggregates in the suspension may shield the signals of smaller particles, leading to an overestimation of measurement values [46,47,48,49].

### 2.4. Stability

**Figure 3:**
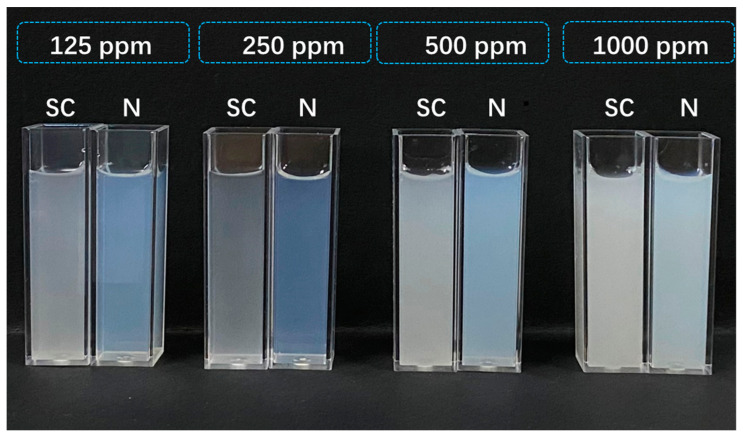
Appearance of the chlorantraniliprole nanosuspension(N) and SC diluted to different concentrations.

As shown in Figure 3, diluted solutions of the chlorantraniliprole nanosuspension at different concentrations exhibited a light blue color due to the Tyndall effect, whereas the SC was milky white at high concentrations. The storage stability of the nanosuspension was further investigated according to CIPAC MT 39, CIPAC MT 46, and GB/T 19136–2003. As shown in Table 3, the average particle size of the nanosuspension increased from 161.9 nm to 182.5 nm and 182.3 nm after 14 days of storage at 4 °C and 54 °C, respectively. However, the particle size changed only slightly when stored at 25 °C. Meanwhile, the PDI value remained below 0.2 during storage. This indicates that the nanosuspension had an excellent physical stability, which can be attributed to the strong electrostatic repulsion combined with the steric stabilization provided by the two polymer surfactants [50,51]. Therefore, reducing the particle size of chlorantraniliprole to the nanoscale is an effective way to improve its stability and shelf life.

### 2.5. Foliar Wettability and Retention

**Figure 4:**
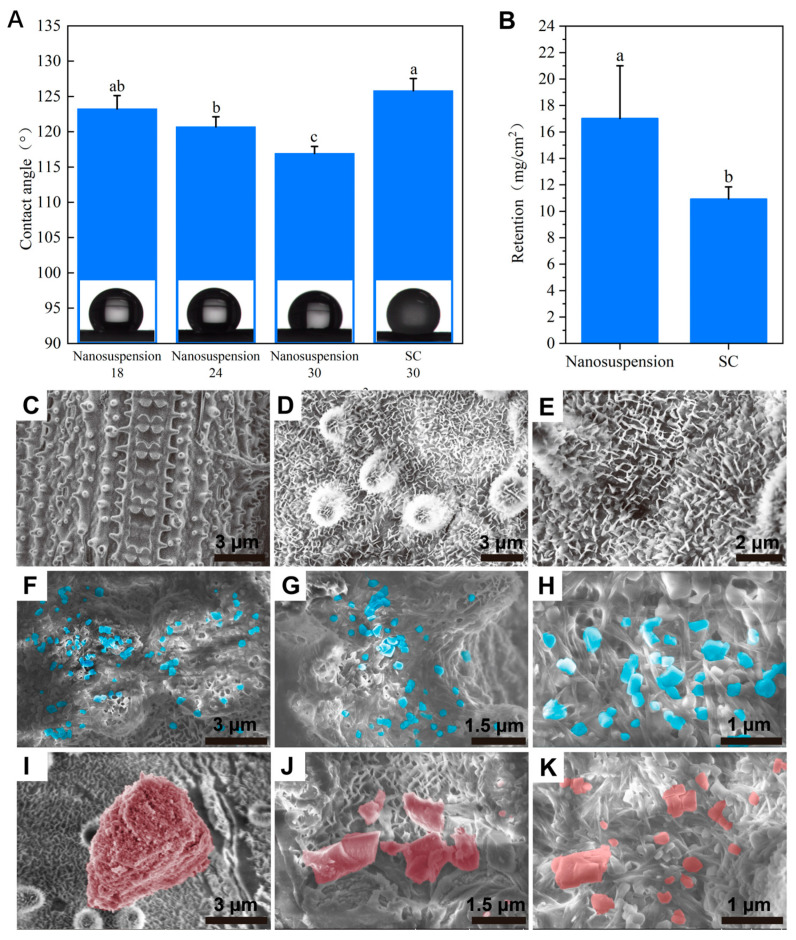
(**A**) Contact angles and (**B**) retention of the chlorantraniliprole nanosuspension and SC on rice leaves. (**C**–**E**) Morphology of blank rice leaves. Depositions and distributions of the chlorantraniliprole particles in (**F**–**H**) the nanosuspension and (**I**–**K**) the SC on rice leaves. Different letters (a,b,c) in the figure indicate significant differences at *p* < 0.05.

The formulations’ foliar wettabilities and retention influence the pesticide efficacy by affecting spreading, adhesion, and droplet persistence on crop leaves after spraying [52,53]. Under different application scenarios, the pesticide formulations will be diluted to different concentrations, so the foliar wettabilities of different diluents of the chlorantraniliprole nanosuspension on rice leaf surfaces were investigated. Hydrophobic leaves such as rice leaves are more difficult to wet and cannot retain water as well as hydrophilic leaves, so it is practical to enhance the pesticide utilization rate on hydrophobic surfaces. As shown in Figure 4A, the contact angle of the chlorantraniliprole SC on a rice leaf was 125.8°, which was comparable to the 18 g a.i./hm^2^ dispersion of the nanosuspension. However, the contact angle of the nanosuspension dispersion continued to decrease with increasing pesticide concentrations, dropping to 120.7° at 24 g a.i./hm^2^ and 116.9° at 30 g a.i./hm^2^, significantly lower than the contact angle of the SC. A reduction in the contact angle is conducive to increasing the foliar wettability of pesticide droplets, which further enhances retention performance. As shown in Figure 4B, the retention of the chlorantraniliprole nanosuspension on rice leaves was 55.9% higher than that of the SC. In order to explore the reasons for the differences in deposition performance, the particle depositions and distributions of the two formulations on the rice leaf surface were observed using a scanning electron microscope. As shown in Figure 4C–E, there were abundant papillae and micro/nanostructures on the rice leaf surface, with widely distributed grooves. This microscopic morphology is more favorable for nanoparticle deposition. Figure 4F–K further confirm the above speculation. Chlorantraniliprole nanoparticles were easily distributed between the grooves, which could effectively inhibit the loss of active ingredients. In contrast, the large particles in the SC could not match the micro/nanostructures of the leaf surface, thus covering the top surface in a blocky form which can easily roll off. Therefore, the higher retention of the chlorantraniliprole nanosuspension can be attributed to its small size and enhanced foliar wettability, facilitating an improvement in the pesticide utilization rate and efficacy.

### 2.6. Field Efficacy on Cnaphalocrocis Medinalis

**Figure 5:**
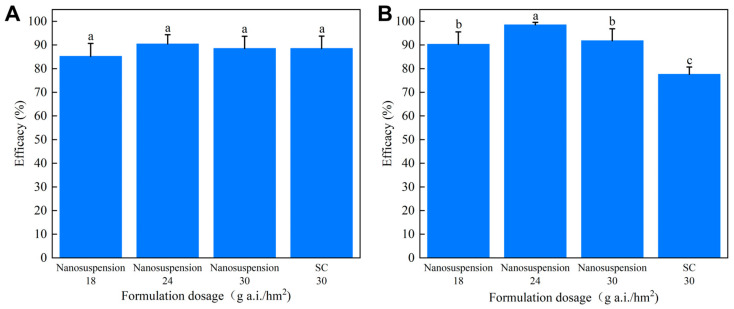
Field efficacies of the chlorantraniliprole nanosuspension and the SC against *Cnaphalocrocis medinalis* spraying using (**A**) a knapsack sprayer and (**B**) a drone. Different letters (a,b,c) in the figure indicate significant differences at *p* < 0.05.

At present, knapsack sprayers still are the most common agricultural machinery used for pest control in China. However, in recent years, with the reduction in the rural workforce and a sharp increase in labor costs, coupled with the increase in agricultural intensification, plant protection drones have been widely used. Limited by low loading capacity, highly concentrated and dispersed pesticide formulations are more suitable for drone spraying. Hence, in this study, field efficacy evaluations of the chlorantraniliprole nanosuspension against *Cnaphalocrocis medinalis* were performed using two different spray modes: a knapsack sprayer and a drone. Under manual spraying, both the nanosuspension and the conventional SC achieved around 85% control efficacies against *Cnaphalocrocis medinalis* (Figure 5A). There was no significant difference in the control effect of the nanosuspension at 18 g a.i./hm^2^ and SC at 30 g a.i./hm^2^ dosages. A lower spraying height in manual mode reduced the influence of the external environment on pesticide droplet adhesion, thereby reducing the differences in efficacy between the various formulations. As shown in Figure 5B, when a drone was used for spraying, the nanosuspension efficacies were greater than 90% at application doses of 18–30 g a.i./hm^2^, but the control effect of the SC was below 80%, which meant that the nanoformulation could reduce pesticide usage by about 40%. In high-concentration spraying, the better dispersibility and suspensibility of the nanosuspension were more conducive to improving the coverage, adhesion, and deposition of active ingredients on crop leaves, thus increasing the contact probability between pesticides and pests and further improving the biological control effect. Conversely, the large particles in the SC were more likely to agglomerate and settle, resulting in concentration discrepancies during the application process and inconsistent control against *Cnaphalocrocis medinalis*. It is evident that chlorantraniliprole nanoformulation use led to a substantial reduction in pesticide usage and increased the biocontrol efficiency, thereby increasing the formulation’s ecological safety. This study provides a scientific basis and data-based support for the development and application of nanopesticides.

### 2.7. Pesticide Residue

Pesticide residues in crop tissues and harvested fruits have a significant effect on the ecological environment and food safety. Table 4 compares the chlorantraniliprole residues on rice leaves and grains after applying the nanosuspension and the SC with the same active ingredient dosage. The maximum residue limits (MRLs) for chlorantraniliprole in rice vary according to countries and regions, with MRLs of 0.5 mg/kg, 0.4 mg/kg, 0.4 mg/kg, 0.02 mg/kg in China, Codex Alimentarius Commission (CAC), Australia, and Europe, respectively. The application of the chlorantraniliprole nanosuspension had low residue risks, thus ensuring food and ecosystem safety. Moreover, considering the field control efficacy, the chlorantraniliprole nanosuspension facilitated a reduction in pesticide usage, which further enhances the formulation’s environmental friendliness, delays pest resistance, and improves the economic benefits.

### 2.8. Impact on Rice Quality

The content and distribution of major nutritional components in rice are pivotal indicators of rice quality. Table 5 shows the effects of the different chlorantraniliprole formulations on the nutrient content in rice grains. Compared to the control group, chlorantraniliprole application had no effect on protein content. Khan et al.’s study also showed that five insecticides—Lorsban (40% EC), Decis (25% EC), Pyrifos (40% EC), Karate (25% EC), and Ripcord (10% EC)—did not have any significant effect on the protein content of chickpea (*Cicer arietinum* L.) crops [54]. However, pesticide application reduced the contents of energy, fat, and carbohydrates, as well as calcium, magnesium, iron, and potassium elements in rice grains. Reddy et al. investigated the influence of insecticide spraying on mineral element contents in cabbage. They found that pesticides significantly reduced the zinc content but increased the calcium and potassium contents. Additionally, endosulfan had a more pronounced effect on altering mineral element contents compared to malathion [55]. The probable reason for this is that pesticides, as exotic stressors, cause a reduction in rice stomatal opening, which in turn results in a decrease in photosynthetic rate and organic matter production, thereby reducing the fat and carbohydrates stored in rice gains [56,57,58,59,60]. Khidir clarified that treflan could significantly reduce the amount of total nitrogen, phosphorus, potassium, sodium, iron, zinc, and manganese in wheat [61]. Moreover, the calcium, magnesium, iron, zinc, and potassium contents in gains treated with nanosuspension were all higher than those in the group sprayed with SC. The nanosuspension has a smaller and more uniform particle size, and may have a lower impact on stomatal opening and photosynthesis. Rice transports mineral elements to plant tissues via the chelation of deoxymalturonic acid (DMA) and mineral elements [62]. Pesticides lost to the soil may also affect the dissolution and chelation of mineral elements, thereby affecting their delivery [63]. In conclusion, the nanosuspension has a smaller impact on the quality of rice. 

### 2.9. Zebrafish Safety

**Figure 6:**
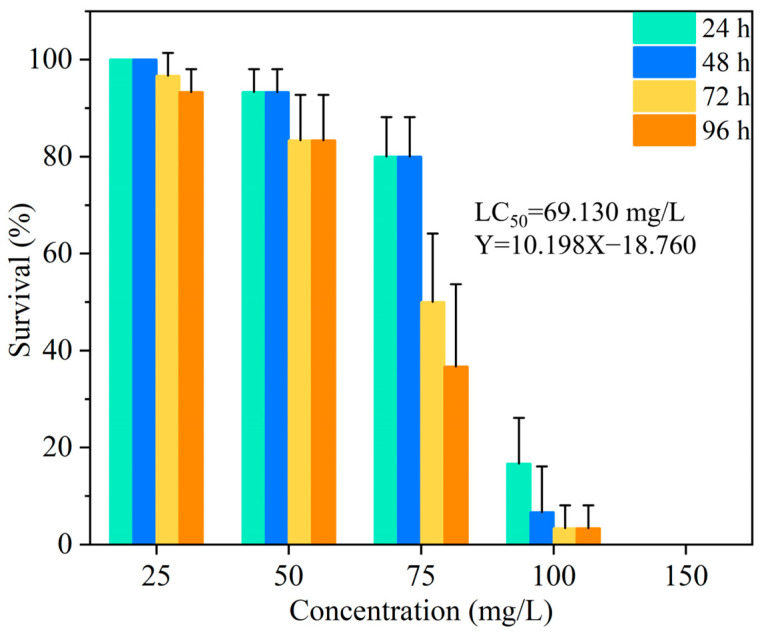
Toxicity of the chlorantraniliprole SC to zebrafish.

Pesticides can enter the aquatic environment of rice paddies and cause serious ecological and environmental problems. In this study, the safety of chlorantraniliprole regarding aquatic organisms was evaluated using zebrafish as a model species. As shown in Figure 6, the mortality of zebrafish increased with increasing chlorantraniliprole concentrations and treatment durations. The LC_50_ value of the chlorantraniliprole SC was 69.13 mg/L, consistent with what is described in [64]. In contrast, all zebrafish survived at each corresponding concentration in the chlorantraniliprole nanosuspension group, even at 500 mg/L. Considering that it would be difficult to reach such high chlorantraniliprole concentrations in the natural environment, it can be inferred that the nanosuspension is extremely safe for zebrafish. An analogous phenomenon has also been noted by other researchers. Huang et al. compared the acute toxicities of three chlorantraniliprole formulations to zebrafish embryos. The LC_50_ of the aqueous solution was greater than 80 mg/L, while the LC_50_ values of the SC and granules were 32.34 mg/L and 25.96 mg/L, respectively [65]. The significant difference in toxicity to zebrafish between the different formulations is mainly linked to the type and content of organic solvents and surfactants in the compositions. In this study, the low toxicity of the chlorantraniliprole nanosuspension was due to chlorantraniliprole’s action mechanism, which interferes with the nervous system of pests to cause paralysis or death [66]. Essentially, chlorantraniliprole is an insensitive agent to zebrafish. In particular, the processing stages and the nanosuspension composition are free of organic solvents and harmful adjuvants, thus rendering it environmentally friendly and safe for non-target organisms.

## 3. Materials and Methods

### 3.1. Materials

The chlorantraniliprole technical material (TC, 95%, *w*/*w*) and suspension concentrate (SC, 200 g/L) were acquired from FMC Corporation (Shanghai, China). Maleic rosinpolyoxypropylene-polyoxyethylene ether sulfonate (MRES) and polycarboxylate were provided by Sinvochem S&D Co., Ltd. (Yangzhou, Jiangsu, China). Chromatographic-grade acetonitrile was bought from Thermo Fisher Scientific Co., Ltd. (Shanghai, China). Milli-Q water (18 MΩ cm, TOC ≤ 4 ppb) was used in all analytical experiments.

### 3.2. Preparation of Chlorantraniliprole Nanosuspension

In our previous work, it was proven that a composite surfactant consisting of MRES and polycarboxylate (1:1, *w*/*w*) is a suitable surfactant system for the prevention of chlorantraniliprole nanoparticle aggregation [67]. Based on this, the same surfactant combination was applied to stabilize a chlorantraniliprole nanosuspension produced via the wet media milling method. The specific preparation procedure was as follows. Firstly, 5.62 g of MRES and the same amount of polycarboxylate were dissolved in 154 mL of water to obtain a colorless and transparent solution. Subsequently, 22.5 g of chlorantraniliprole was added into the above solution, and the mixture was stirred and emulsified at rcf = 1453 g for 10 min in a shearing machine (25BC, Shanghai HENC Mechanical Equipment Co., Ltd., Shanghai, China) to uniformly suspend the pesticide particles in the suspension. Then, the dispersion was transferred to laboratory milling equipment (WG-0.3, Suzhou Vgreen Nano-Chem Technology Co., Ltd., Suzhou, Jiangsu, China) to produce chlorantraniliprole nanosuspensions. The milling media were 0.3 mm zirconium dioxide beads. The surfactant content and milling time changed with the experimental design.

### 3.3. Particle Size and Zeta Potential Measurements

The chlorantraniliprole nanosuspension and SC were diluted with water and then placed into the sample cells of a Zetasizer Nano ZS 90 (Malvern, UK). The particle size, polydispersity index (PDI), and zeta potential were measured at room temperature. Each sample was measured in triplicate, and the data were recorded as means ± standard deviation (S.D.).

### 3.4. Morphological Characterization

The morphological characterization of the chlorantraniliprole particles was performed using a scanning electron microscope (JSM-7401F, JEOL, Tokyo, Japan). Aqueous dispersions of the chlorantraniliprole nanosuspension and SC were dropped onto freshly cleaned silicon slices. The samples were air-dried and coated with platinum using a sputter coater (ETD-800, Beijing Elaborate Technology Development Ltd., Beijing, China). The images were recorded in low electron image (LEI) mode, and the statistical particle sizes were determined based on SEM images using Nano Measurer software (1.2.5).

### 3.5. Stability Test

The physical stability of the chlorantraniliprole nanosuspension was assessed according to CIPAC MT 39, CIPAC MT 46, and GB/T 19136–2003. The samples were stored at 0 ± 2 °C, 25 ± 2 °C, and 54 ± 2 °C for 14 days. The particle size and PDI were measured every two days.

### 3.6. Contact Angle Measurement

The contact angles of the chlorantraniliprole nanosuspension and SC on rice leaves were measured using a contact angle apparatus (JC2000D, Zhongchen Digital Technic Apparatus Co., Ltd., Shanghai, China). The samples were diluted with water to different concentrations corresponding to those applied in the field. Fresh rice leaves were smoothly adhered to glass slides. Then, a drop of the sample aqueous dispersion was placed onto the surface of the rice leaf. The droplet was photographed after standing for 15 s, and the five-point fitting method was used to calculate the contact angle. The average value of five replicates was calculated. 

### 3.7. Retention Test

The retention was measured (R_m_, mg/cm) according to the method described in [68] with slight modifications. First, the chlorantraniliprole nanosuspension and SC were diluted into aqueous dispersions containing 0.1% (*w*/*w*) of active ingredient. Secondly, each leaf was weighed using an electronic balance (ME204E, Mettler Toledo, Zurich, Switzerland), and its surface area was measured using a leaf area meter (Yaxin-1241, Beijing Yaxin Science Instrument Technology Co., Ltd., Beijing, China). Leaves were then completely immersed in the above dispersions. After 15 s, each leaf was removed and weighed again. The retention (R_m_) was calculated based on the following equation. The particle adsorption and distribution of the chlorantraniliprole nanosuspension and SC on rice leaves were also observed using a scanning electron microscope.

(1) $$R=\frac{M_{1}-M_{0}}{S}$$

where M_0_ (mg) and M_1_ (mg) are the leaf weights before and after immersion in dispersions, and S (cm^2^) is the leaf area. The average of five tests was calculated.

### 3.8. Field Efficacy on Cnaphalocrocis Medinalis

The field efficacies of the chlorantraniliprole nanosuspension and SC with different size characteristics were compared. The field trials for the control effect on *Cnaphalocrocis medinalis* were performed at two sites. A knapsack sprayer was used to spray pesticide in Baiertu Village, Lubu Town, Zhaoqing City, Guangdong Province, China, from 10 to 25 May 2019, while a drone was applied for spraying at Mawang Village, Wuling Town, Bingyang County, Nanning City, Guangxi Province, China, from 2 to 17 June 2019. Each experimental area was 667 m^2^ and arranged in a randomized block design. The concentration of the chlorantraniliprole SC was set as 30 g a.i./hm^2^, considering the recommended dosage. The nanosuspension was diluted into three concentration gradients of 18, 24, and 30 g a.i./hm^2^. The parallel skip method was used for sampling, and 50 samples were taken from each treatment area. Three replicates were conducted for each treatment.

### 3.9. Residue Test

#### 3.9.1. Sample Collection and Processing

Rice leaf samples were collected 21 days after pesticide application, and grain samples were collected after the rice was mature. Samples were gathered independently from at least 12 random sites in each plot. After collection, 2.000 g of accurately weighed rice leaves (cutting into pieces less than 1 cm) or crushed grains was placed into 50 mL centrifuge tubes. Subsequently, 19.8 mL of acetonitrile, 0.2 mL of acetic acid, 1.5 g of anhydrous sodium acetate, and 6 g of anhydrous magnesium sulfate were added and homogenized at RCF = 503 g for 1 min, followed by centrifugation at RCF = 905 g for 5 min. Then, 6 mL of the upper acetonitrile extract was added into a centrifuge tube filled with 900 mg of anhydrous magnesium sulfate and 150 mg of PSA (primary secondary amine) sorbent. After vortex mixing for 1 min and centrifugation at RCF = 905 g for 5 min, 0.75 mL of the supernatant was mixed with water in a 1:1 ratio (*v*/*v*) and passed through a 0.22 μm filter membrane for testing. The same method was used to determine the chlorantraniliprole recovery rate. Three concentration levels (10 μg/L, 20 μg/L, and 30 μg/L) were used for the recovery test, and three parallel tests were conducted for each sample.

#### 3.9.2. Chromatographic Analysis Conditions

Liquid phase separation was performed by gradient elution using a Kinetex C18 column (4.6 mm × 100 mm, 2.6 μm) under the conditions listed in Table 6. The analysis time for each sample was 7 min. The mobile phase consisted of water (with 1‰ formic acid) and acetonitrile. The column temperature was maintained at 25 °C.

#### 3.9.3. Mass Spectrometric Analysis Conditions

Chlorantraniliprole was detected in positive-ion mode because of the minimal background interferences and high response. The protonated molecular ion peak [M + H]^+^ of chlorantraniliprole was observed at *m*/*z* 484. The [M + H]^+^ ion was selected as the precursor ion for secondary mass spectrometry analysis, and the major fragmentation ions of chlorantraniliprole were located at *m*/*z* 453 and 286. These fragmentation ions exhibited strong and stable responses, thus acting as product ions. An automated optimization approach was employed to maximize the response of the target compound under multiple reaction monitoring (MRM) mode, ensuring the highest sensitivity to analytes.

#### 3.9.4. Measurement of Additive Recovery

The standard curve equation of chlorantraniliprole, measured by liquid chromatography–mass spectrometry, was Y = 8020.2X + 835.67, with a correlation coefficient R2 of 0.9999. This indicates that there was a good linear relationship between the concentration of chlorantraniliprole and the peak area in the range of 0.5–50 μg/L. A recovery test at three chlorantraniliprole concentrations was carried out, and the average recovery rate of the added standard concentrations of 10 μg/L, 20 μg/L, and 30 μg/L ranged from 80.1 to 109.63%. The results in Table 7 show that the RSD of the three measurements is 0.9–4.2%, indicating good recovery and reproducibility.

### 3.10. Rice Quality Determination

The protein content in rice was determined using the Kjeldahl nitrogen method according to GB 5009.5-2016. In detail, 3 g of rice was taken, and under catalytic heating conditions, proteins were decomposed, and the produced ammonia was combined with sulfuric acid to form ammonium sulfate. Alkaline distillation was performed to release ammonia, which was then absorbed by boric acid and titrated using a standard sulfuric acid solution. The nitrogen content was calculated based on the consumption of acid and multiplied by the conversion factor to obtain the protein content. The determination of fat in rice was carried out using the Soxhlet extraction method following GB 5009.6-2016. For this, 3 g of rice was accurately weighed and placed in a filter paper tube. The tube was then put into a Soxhlet extractor with anhydrous ether, and the mixture was heated under reflux. After drying, the fat mass was measured. The calculation of the energy and total carbohydrates in rice was based on GB 28050-2011. The total carbohydrates were calculated as the total of food mass minus the mass of protein, fat, water, and ash. The energy content was calculated by multiplying the contents of protein, fat, and total carbohydrates by energy factors of 17, 37, and 17 kJ/g, respectively, and then summing them up per 100 g of the product. The determination of mineral trace elements in rice was carried out using the Inductively Coupled Plasma Atomic Emission Spectrometry (ICP-AES) method following GB 5009.268-2016. After the 3 g rice sample was digested, it was measured using an inductively coupled plasma atomic emission spectrometer. The qualitative analysis was based on the characteristic spectral line wavelength of the element, and the quantitative analysis was performed using the direct proportionality between the signal intensity of the element spectral line and its concentration.

### 3.11. Toxicity against Zebrafish

The chlorantraniliprole nanosuspension and SC were diluted with water to obtain sample suspensions at several different concentrations. Ten adult zebrafish were randomly selected and transferred to the sample suspensions. During the period of the experiment, the suspensions were replaced every 24 h to maintain the pesticide concentration and water quality. All zebrafish were reared at a temperature of 25 ± 1 °C. The poisoning symptoms and mortality of the tested zebrafish within 24, 48, 72, and 96 h were observed. The judgment standard for death status was no breathing or no movement upon touching the tail.

### 3.12. Statistical Analysis

Data were analyzed using a one-way analysis of variance (ANOVA) and Duncan’s multiple range tests, and a significance level of less than 0.05 was considered statistically significant.

## 4. Conclusions

In this study, a chlorantraniliprole nanosuspension with an average particle size of 56 nm and excellent stability was produced by using wet media milling technology, providing a green and scalable strategy to construct nano-delivery systems for pesticides that are poorly soluble in both water and organic solvents. Compared to the SC formulation, the nanosuspension exhibited better dispersibility, foliar wetting, and retention. The field trials confirmed that pesticide usage could be reduced by about 40% through using the nanoformulation while maintaining the same level of efficacy as the SC. It is worth noting that the nanosuspension exhibited lower residual properties and higher non-target biosafety while exerting a high insecticidal activity, reducing the negative impact of pesticides on rice and ensuring rice quality and food safety. These advantages are attributed to the nanosuspension’s environmentally friendly composition and processing procedure. Comprehensive efficacy and safety evaluations of chlorantraniliprole nanosuspensions are of paramount importance for guiding the design, construction, and application of nanopesticides. This highly effective and eco-friendly nanosuspension has broad application prospects in crop protection for improving pesticide efficacy and reducing residual pollution in agricultural products and the environment.

## Figures and Tables

**Table 1:** Effects of surfactant content and milling time on the particle size and distribution of the chlorantraniliprole nanosuspensions.

		Size (nm)	PDI
Surfactant/pesticide ratio	30%	167.1 ± 1.2	0.174 ± 0.013
	50%	161.9 ± 0.2	0.186 ± 0.010
	70%	160.3 ± 0.8	0.153 ± 0.007
Milling time (h)	0.5	288.4 ± 2.9	0.253 ± 0.009
	1	244.2 ± 3.1	0.174 ± 0.024
	2	161.9 ± 0.2	0.186 ± 0.010
	3	181.0 ± 1.4	0.176 ± 0.015

**Table 2:** Particle size, distribution, and zeta potential values of the chlorantraniliprole nanosuspension and SC.

Formulation	Size (nm)	PDI	Zeta Potential (mV)
Nanosuspension	161.9 ± 0.2 b	0.186 ± 0.010 b	−43.1 ± 0.7 b
SC	678.7 ± 27.3 a	0.446 ± 0.021 a	−29.4 ± 0.3 a

Different letters (a,b) in the table indicate significant differences at *p* < 0.05.

**Table 3:** Storage stability of the chlorantraniliprole nanosuspension at 4 °C, 25 °C, and 54 °C.

Time (Day)	4 °C		25 °C		54 °C	
	Size (nm)	PDI	Size (nm)	PDI	Size (nm)	PDI
0	161.9 ± 0.2 e	0.186 ± 0.010 a	161.9 ± 0.2 bc	0.186 ± 0.010 a	161.9 ± 0.2 e	0.186 ± 0.010 a
2	166.1 ± 0.6 d	0.143 ± 0.006 e	159.0 ± 1.3 c	0.141 ± 0.008 b	156.7 ± 0.5 f	0.163 ± 0.004 ab
4	166.4 ± 0.6 d	0.137 ± 0.005 e	162.9 ± 1.5 bc	0.134 ± 0.008 b	173.9 ± 1.3 c	0.167 ± 0.002 ab
6	176.1 ± 0.4 c	0.159 ± 0.005 cd	164.6 ± 2.8 ab	0.147 ± 0.003 b	169.4 ± 0.3 d	0.149 ± 0.007 bc
8	175.8 ± 0.2 c	0.166 ± 0.011 bc	169.3 ± 3.2 a	0.146 ± 0.019 b	169.8 ± 0.7 d	0.133 ± 0.012 cd
10	177.5 ± 1.0 bc	0.148 ± 0.009 de	167.9 ± 2.6 a	0.151 ± 0.012 b	178.3 ± 2.5 b	0.165 ± 0.010 ab
12	178.8 ± 1.4 b	0.174 ± 0.007 ab	166.9 ± 2.6 ab	0.141 ± 0.008 b	176.4 ± 0.7 b	0.117 ± 0.023 d
14	182.5 ± 0.4 a	0.168 ± 0.007 bc	166.1 ± 0.8 ab	0.134 ± 0.017 b	182.3 ± 0.3 a	0.168 ± 0.012 ab

Different letters (a,b,c,d,e,f) in the table indicate significant differences at *p* < 0.05.

**Table 4:** Residues of chlorantraniliprole in rice treated with the proposed nanosuspension and SC.

Sample	Treatment	Residue Amount (mg/kg)
Leaves	SC 30 g a.i./hm^2^	0.074 ± 0.0021
	nanosuspension 30 g a.i./hm^2^	0.063 ± 0.0006
Grains	SC 30 g a.i./hm^2^	0.042 ± 0.0006
	nanosuspension 30 g a.i./hm^2^	Not detected

**Table 5:** Effects of applying the chlorantraniliprole nanosuspension and the SC on rice quality.

Nutrient	Control Group	Nanosuspension	SC
Energy	1550 kJ/100 g	1511 kJ/100 g	1498 kJ/100 g
Protein	7.2 g/100 g	7.2 g/100 g	7.2 g/100 g
Fat	1.4 g/100 g	0.9 g/100 g	0.9 g/100 g
Carbohydrate	80.0 g/100 g	78.6 g/100 g	77.8 g/100 g
Dietary fiber	2.0 g/100 g	2.4 g/100 g	2.5 g/100 g
Calcium	89.4 mg/kg	88.2 mg/kg	81.7 mg/kg
Magnesium	587 mg/kg	556 mg/kg	413 mg/kg
Iron	7.82 mg/kg	6.21 mg/kg	5.41 mg/kg
Zinc	21.4 mg/kg	23.1 mg/kg	19.6 mg/kg
Potassium	1630 mg/kg	1480 mg/kg	1170 mg/kg

**Table 6:** Mobile phase parameters for gradient elution in liquid chromatography.

Time (min)	Flow Rate (mL/min)	Water Phase	Acetonitrile
0	0.6	60	40
0.5	0.6	60	40
5	0.6	5	95
5.5	0.6	5	95
6	0.6	60	40
7	0.6	60	40

**Table 7:** Recovery rate of chlorantraniliprole addition in rice.

Treatment	Number	10 μg/L	20 μg/L	30 μg/L	RSD (%)
Chlorantraniliprole (leaf)	1	79.6%	89.7%	109.2%	0.9
	2	78.4%	87.6%	107.4%	
	3	82.3%	85.4%	112.3%	
	average	80.10%	87.57%	109.63%	
Chlorantraniliprole (grain)	1	80.2%	85.6%	105.9%	4.2
	2	78.9%	84.3%	107.3%	
	3	84.1%	80.1%	99.6%	
	average	81.07%	83.33%	104.20%	

## Data Availability

Research data are available upon request to the authors.
